# Protein-Containing Breakfasts Are Associated with Reduced Postprandial Glycaemic Excursions in Children and Young People with Type 1 Diabetes: A Pragmatic Study

**DOI:** 10.3390/nu18152536

**Published:** 2026-08-04

**Authors:** Julie Johnson, Victoria Franklin, Ashley Shepherd, Sonja Allen, Debbie Blissitt, Kate Keen, Kirsty Maclean, Anne-Marie McKillup, Elizabeth Procter, Susan Roach, Adele Swart, Stuart D. R. Galloway

**Affiliations:** 1 Department of Nutrition and Dietetics, NHS Highland, Raigmore Hospital, Inverness IV2 3UJ, UK; 2 Faculty of Health Sciences and Sport, University of Stirling, Stirling FK9 4LA, UK; ashley.shepherd@stir.ac.uk (A.S.); s.d.r.galloway@stir.ac.uk (S.D.R.G.); 3 Department of Paediatrics, NHS Highland, Raigmore Hospital, Inverness IV2 3BW, UK; victoria.franklin@nhs.scot; 4 Department of Paediatric Diabetes, Croydon University Hospital, 530 London Road, Thornton Heath CR7 7YE, UK; sonja.allen3@nhs.net; 5 Department of Nutrition and Dietetics, The Hillingdon Hospital NHS Foundation Trust, Pield Heath Road, London UB8 3NN, UK; debbie.blissitt@nhs.net; 6 Department of Nutrition and Dietetics, Epsom & St Helier University Hospitals NHS Trust, Wrythe Lane, London SM5 1AA, UK; k.keen@nhs.net; 7 Department of Nutrition and Dietetics, NHS Greater Glasgow and Clyde, Royal Hospital for Children, 1345 Govan Road, Govan, Glasgow G51 4TF, UK; kirsty.maclean9@nhs.scot; 8 Department of Nutrition and Dietetics, Evelina London Children’s Hospital, St Thomas’ Hospital, Westminster Bridge Road, London SE1 7EH, UK; annemarie.mckillup@nhs.net; 9 Department of Nutrition and Dietetics, Queens Medical Centre, Nottingham University Hospitals NHS Trust, Derby Road, Nottingham NG7 2UH, UK; elizabeth.procter5@nhs.net; 10 Department of Nutrition and Dietetics, Leicester Royal Infirmary, University Hospitals of Leicester NHS Trust, Infirmary Square, Leicester LE1 5WW, UK; 11 Department of Paediatric Diabetes, Kaleidoscope CYP Centre, Lewisham and Greenwich NHS Trust, 32 Rushey Green, London SE6 4JF, UK; adele.swart@nhs.net

**Keywords:** carbohydrate, continuous glucose monitoring, diabetes mellitus, glycaemic control, meal, nutrition, protein

## Abstract

**Background/Objectives**: This study examined whether adding 10 g of protein to a high glycaemic index (GI) breakfast could attenuate postprandial glycaemic responses in children and young people (CYP) with type 1 diabetes (T1D). Postprandial glucose responses following this modified breakfast were compared with those after a high GI breakfast alone, a low GI breakfast, and participants’ usual breakfast. **Methods**: A pragmatic randomised crossover study was conducted in *n* = 25 CYP aged 5–17 years. Participants consumed three standardised test breakfasts on two study occasions and their usual breakfast as a control. Test meals varied by GI, glycaemic load (GL), and protein content: high GI/high GL (HGL), high GI/high GL with an additional 10 g protein (HGLP), and low GI/medium GL (MGL). Continuous glucose monitoring data were collected for three hours following each meal. Linear mixed model analyses were used, and a subgroup analysis included participants using hybrid closed-loop (HCL) systems. **Results**: Participants’ (mean age 12.1 ± 3.6 years) mean postprandial glucose over a three-hour follow-up was significantly lower following the HGLP meal compared with the HGL meal (8.0 ± 2.2 vs. 9.5 ± 2.5 mmol/L; *p* < 0.01). Time in Range (TIR) over 180 min was significantly shorter after the HGL meal compared with the control, HGLP, and MGL meals (*p* ≤ 0.03). Glucose excursions at 30 and 60 min were significantly higher following the HGL meal compared with all other meals, with differences persisting up to 120 min when compared with the HGLP meal. Results were not different for participants using HCL systems. **Conclusions**: Adding 10 g of protein to a high GI breakfast or choosing a lower GI option improves postprandial glycaemic control in CYP with type 1 diabetes.

## 1. Introduction

Carbohydrate is the primary macronutrient influencing postprandial glycaemia in T1D, and dietary management is underpinned by quantifying carbohydrate intake and matching insulin dosing accordingly [1]. However, even with accurate carbohydrate counting, postprandial hyperglycaemia remains common in CYP with T1D and is particularly pronounced after breakfast compared with other meals [2,3,4]. This exaggerated breakfast response is multifactorial but is strongly influenced by the composition of commonly consumed breakfast foods [5].

Ready-to-eat breakfast cereals are widely consumed by CYP in the UK [6]. While these foods may contribute to fibre and micronutrient intake [7,8,9], many have a high glycaemic index (GI) and glycaemic load (GL) [10] and are associated with pronounced postprandial glucose excursions [11,12]. Substitution of high-GI foods with lower-GI alternatives is recommended within the International Society of Paediatric and Adolescent Diabetes (ISPAD) nutritional guidelines; these guidelines also suggest modifying breakfast meals prone to postprandial hyperglycaemia with the addition of protein [1].

Dietary protein has been shown to modify postprandial glycaemic responses in T1D [13,14,15]; however, the ISPAD guidelines [1] do not specify the quantity of protein required to meaningfully alter the glucose response to breakfast, highlighting an important evidence gap. Moreover, research examining protein-mediated modulation of postprandial glycaemia in T1D has largely focused on high-fat and high-protein meals, where delayed hyperglycaemia and increased insulin requirements are observed [13,15,16,17,18]. Breakfast-specific responses, particularly in the context of high GI meals, remain under-investigated.

In healthy adults, the addition of a protein food containing 30 g protein and 7 g fat to a high-GI breakfast has been shown to significantly reduce the GI of the meal [19]. In adults with T1D, the addition of protein and fat in the form of peanut butter to a high- and low-GI meal of equal macronutrient composition produced no significant difference in glycaemic response between these meals, suggesting the protein and/or fat altered the GI of the meal [20]. Protein intakes of 22 g with 9 g fat consumed at breakfast 15 min before a carbohydrate-containing meal have been shown to attenuate early postprandial glucose rises, although short monitoring periods limit conclusions regarding delayed hyperglycaemia [21]. Studies employing longer postprandial observation periods, including those involving CYP with T1D, suggest that small-to-moderate protein intakes (≤28 g) with fat (≤19 g) consumed alongside carbohydrate can attenuate early glycaemic excursions without inducing late hyperglycaemia [13,14,22]. In contrast, larger protein and fat loads (>40 g) have been associated with delayed hyperglycaemia several hours after ingestion [13,14,22,23,24].

Notably, studies examining protein addition, with or without fat, at breakfast have not included high GI/GL breakfast cereal meals, despite their widespread consumption. In our previous research, CYP with T1D were observed to consume a wide variety of breakfast choices, with cereal-based meals the most frequent choice of food [25]. These meals were characterised by high GI and GL values and were associated with the largest postprandial glucose excursions. In contrast, breakfasts containing a protein food were associated with significantly lower glycaemic responses. The mean difference in protein intake between meals with or without a protein food was 10.6 g (95% CI [9.1, 12.1], and these had a total mean protein content of 20.2 ± 9.3 g. Similarly, the mean difference in the fat content of the meals with or without a protein food was 10 g (95% CI [7.8, 12.3] with a mean fat content of 19.2 ± 10.3 g, quantities that would be unlikely to provoke late postprandial hyperglycaemia [25].

Dietary interventions pose substantial logistical and acceptability challenges, often resulting in poorer recruitment and adherence than pharmaceutical trials. These challenges are amplified in paediatric populations, with children and young people (CYP) being under-represented in clinical trials [26], particularly for those living with a chronic condition such as type 1 diabetes (T1D). Low recruitment and retention rates are widely reported across paediatric randomised controlled trials (RCTs), including trials in asthma [27], cancer [28], and T1D [29]. In T1D specifically, increased study complexity, older age, poorer baseline glycaemic control, and lower household income have been associated with reduced recruitment [30]. Poor recruitment limits validity [31] and reduces the translational value of findings to real-world dietary intake and clinical practice [32]. A recent scoping review highlighted a paucity of nutrition research addressing the practical realities of dietary management in paediatric T1D [33]. This underscores the need for pragmatic, clinically relevant dietary research in this population. While observational and pragmatic study designs are more susceptible to confounding than RCTs, they have been shown to produce comparable estimates of treatment effects [34] and offer advantages in capturing real-world dietary practices. Given the challenges inherent in conducting highly controlled nutritional RCTs in paediatric populations, pragmatic approaches provide more clinically meaningful evidence to inform nutritional guidelines.

To date, no studies have directly examined the effect of adding small amounts of a protein food containing both protein and fat to high-GI cereal breakfasts on postprandial glycaemic control in CYP with T1D using a pragmatic approach. Therefore, the aim of this study was to examine the effect of adding a food containing 10 g of protein with varying amounts of fat to a high-GI breakfast and to compare the resulting three-hour postprandial glycaemic response with that of the same breakfast without added protein, and with a low-GI breakfast in children and young people with type 1 diabetes.

## 2. Materials and Methods

This was a pragmatic randomised crossover multicentre study of CYP with T1D who were recruited from 13 diabetes centres in the United Kingdom (UK), with data collection conducted over a one-year period. Eligibility criteria included being aged 5–17 years, having a diagnosis of T1D of at least one year’s duration, and using either MDI or CSII insulin therapy alongside Dexcom CGM (Dexcom, Inc. San Diego, CA, USA). Exclusion criteria comprised the presence of medical conditions that could alter gastrointestinal function or nutrient absorption, including coeliac disease and gastroparesis, as well as the use of medications known to affect glycaemic regulation, such as metformin or antidepressants. Owing to the types of foods in the test meals, vegans and those allergic to any of the food in the test meals were also not eligible to take part.

Recruitment was undertaken by local paediatric diabetes dietitians, who identified eligible CYP and invited them to take part in the study. Participants were asked to consume the test meals and complete a questionnaire evaluating the meals and their postprandial experiences over a 3-h period. At enrolment, diabetes management, including insulin-to-carbohydrate ratios, was reviewed and optimised by the recruiting dietitian or another member of the local diabetes care team as required. Participant information sheets were provided to all families. Written informed consent was obtained from parents or guardians and from participants aged 16–17 years, while participants under 16 years of age provided written assent.

Baseline information was collected at recruitment from clinical records by the recruiting dietitian. This included: sex, date of birth, Body Mass Index (BMI), HbA1c and insulin regimen. Online Surveys (Jisc, Bristol, UK) were used to gather information from participants and included questions about which meal was tested and the diabetes management as well as the postprandial period response. For control meals, the participants were asked to provide information on the meal, including the type and amount of foods consumed.

For the intervention, there were three test meals repeated twice. Two of the test meals consisted of a high GI breakfast cereal, which had a high glycaemic load (HGL) (Supplementary Data, Table S1), and the same HGL meal including a protein food providing 10 g of protein (HGLP) (Supplementary Data, Table S2). In order to reflect popular food choices, there were two choices of cereal (rice snaps or corn flakes) and a choice of protein sources consisting of egg, cheese, or meats, or their combination. Written guidance was provided to the participants detailing portion sizes corresponding to 10 g protein. There were three different portion sizes for the HGL and HGLP meals, providing 35 g, 50 g, and 70 g of carbohydrate to accommodate the different age groups only. The participants chose their own cereal, protein source and portion size but were asked to replicate these exactly when meals were repeated. The protein food was to be consumed alongside other components of the breakfast meal, with no restrictions on timing relative to carbohydrate intake. The final test meal had a low GI with a medium glycaemic load (MGL), which contained 40 g of carbohydrate and consisted of one slice of enriched-fibre white bread with chocolate nut spread plus natural yoghurt and mixed berries (Supplementary Data, Table S3). Participants were asked to complete and submit a questionnaire after consuming the test meal. The questionnaire collected information on the meal consumed, including portion size, cereal type, and protein source, and the 3-h postprandial period enabled assessment of adherence to the dietary instructions.

Participants acted as their own controls, with the control meal being their usual breakfast of choice. The participants were asked to consume each test meal and the control meal in a randomised order on two separate occasions. The randomisation was provided to them using Latin square randomisation [35]. The participants were advised to follow their usual diabetes management for meals, which included the timing of their insulin dose. The participants were also asked to consume >75% of the test meals to ensure this achieved the glycaemic load category and to match consumption on repeat meals.

None of the meals were provided by the research team. As this was a pragmatic real-world study, the participants consumed the meals in their own home on days of their choosing. They were asked to test the meals on days when there had been no nocturnal hypoglycaemia and the CGM sensor reading was between 4 and 10 mmol/L prior to the breakfast meal commencing. During the three-hour postprandial period, participants were also asked to keep physical activity to a maximum of 30 min and to replicate this on all repeat meals. Participants were also asked to avoid consuming any additional food during the postprandial period other than for treating hypoglycaemia. This was to reflect a typical day when snacking often occurs within three hours of the breakfast meal as observed in our observation study [25].

A registered dietitian (JJ) analysed breakfast meals using Nutritics (Nutrition Analysis Software 2019, Nutritics Ltd., Dublin, Ireland). Energy (kcal), fat, saturated fat, carbohydrate, sugars, protein, fibre, salt, GI, and GL were determined, with macronutrient content compared against ISPAD recommendations [1]. GI values were obtained from the International Tables of Glycaemic Index [10,36] or estimated using comparable foods when unavailable. Meal GI was calculated using the method of Dodd et al. (2011) [37], and GL was derived as GI × available carbohydrate (g)/100 [38].

Participants used their own Dexcom G6 or G7 CGM devices during the study, with approval from Dexcom Inc. Data were retrospectively obtained from Dexcom Clarity after each test meal, and meals with ≥70% CGM data availability were included in the analysis [39]. The primary outcome was mean postprandial glucose over 3 h. Secondary outcomes included pre-prandial glucose, timing of insulin administration relative to meal consumption, glucose excursion (mean and peak), time to peak glucose, incremental area under the curve (iAUC), coefficient of variation (CV), TBR (<3.9 mmol/L), TIR (3.9–10.0 mmol/L), TITR (3.9–7.8 mmol/L), and TAR (>10.0 mmol/L) [40]. Post hoc analyses examined differences in glycaemic outcomes between HGLP meals containing <10 g and >10 g total fat.

Data were analysed using Jamovi (Version 2.2.5; The Jamovi Project, Sydney, Australia, 2023). GraphPad Prism version 9.5.0 (GraphPad Software, San Diego, CA, USA) was used for data visualisation and calculation of incremental area under the curve (iAUC) by the trapezoidal method. Results are presented as mean ± SD for normally distributed variables and median (IQR) for non-normally distributed variables. Linear mixed-effects models were used to compare nutrient and glucose outcomes and to evaluate differences in postprandial glucose responses between meal conditions. Analyses were performed for the entire 3-h postprandial period and for consecutive 30-min intervals. Models were fitted using restricted maximum likelihood (REML), with Wald confidence intervals and the Satterthwaite approximation for degrees of freedom. Meal condition was specified as a fixed effect, while participant was included as a random intercept to account for repeated measures within individuals. This modelling approach was chosen because it appropriately accounts for the correlated nature of repeated observations and accommodates unbalanced data resulting from unequal numbers of observations and missing meal measurements. Hypoglycaemic events were analysed using a Generalised Linear Mixed Model with logistic regression. The effect size for the linear mixed models was calculated using the suggested formula from Westfall et al. (2014) [41] (measured as *d*) using the formula applicable for one random factor from Brysbaert & Stevens (2018) [42]. The reliability of repeat meals was analysed via construction of an unconditional Linear Mixed Model to generate the Intraclass Correlation coefficient (ICC), with an ICC of <0.50 judged to show poor reliability [43]. Where appropriate, post hoc tests were performed using the Bonferroni correction to manage the risk of a type 1 error occurring with multiple statistical tests. *p* < 0.05 was considered statistically significant.

Similar previous studies have reported mean differences of 2 mmol/L [14,17,44,45] and a standard deviation of 3.1 mmol/L [46] for blood glucose, giving an effect size of 0.64. With this effect size and statistical power of 80% with alpha set at 0.05, a total sample size of *n* = 22 was required for two-tailed comparisons between two dependent means. We aimed to recruit *n* = 50 to allow for expected high loss to follow-up.

## 3. Results

### 3.1. Participants

Forty-eight participants consented to join the study and provided the baseline information. Of these, five did not respond, and eight withdrew before sharing their CGM data. Of the thirty-five who shared their CGM data, nine did not respond to the request to commence testing the meals, and one started the meals; however, it was not possible to obtain their CGM data. This resulted in 25 participants testing the meals (Table 1).

**Figure 1:**
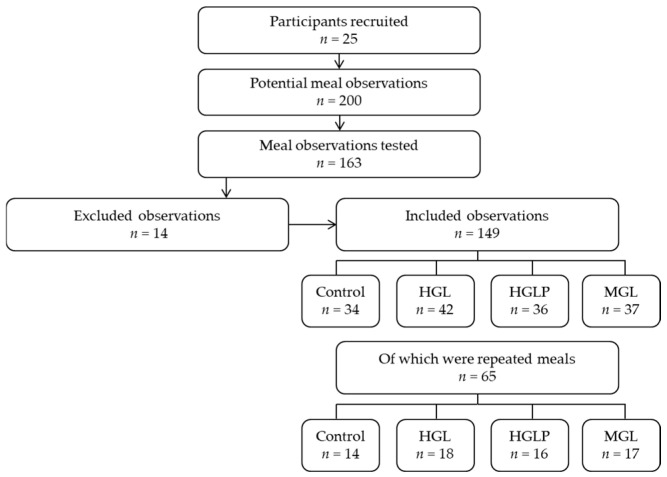
The number of participants who tested meals, repeated meals, and the total number of each type of meal included in the analysis. HGL (high glycaemic load), HGLP (high glycaemic load with added protein), and MGL (moderate glycaemic load).

Fourteen meal submissions were excluded. The reasons for exclusions were: pre-meal sensor reading did not meet protocol conditions (6 meals), duplicate submissions (3 meals), no CGM data available or CGM reported to be inaccurate (2 meals), family reported incorrect insulin dose given (2 meals), and incomplete information (1 meal). The twenty-five participants provided a potential meal observation of 200 meals; however, some participants did not complete or submit questionnaires on all the meals. A total of 149 meals were analysed (Figure 1).

### 3.2. Meal Choice and Composition

The majority of chosen meals were rice cereals of small portion size (Supplementary Data, Table S4). Chosen protein sources were egg, cheese, or meat, or their combination (Supplementary Data, Table S5). There was only one choice of meal for the MGL meal (Supplementary Data, Table S3). The carbohydrate content of the control meals was significantly higher than the HGLP (*p* = 0.02) and MGL meals (*p* = 0.002); however, there was no significant difference in the mean carbohydrate content of the three types of test meals (*p* > 0.05) (Supplementary Data, Table S6). The mean protein of the HGLP meal was 18.5 ± 1.6 g. As the participants could choose from a selection of protein foods, the amount of fat in the HGLP meals varied. Many of the participants chose similar protein sources, and 58.3% of the HGLP meals had a fat content of between 12 and 13 g. None of the HGLP meals contained more than 20 g of fat. There was no significant difference in the mean postprandial glucose over the 180 min after ingestion of the HGLP meals containing less than 10 g fat (mean: 7.5 ± 2.1 mmol/L) compared with the HGLP meals containing more than 10 g of fat (mean: 8.1 ± 2.2 mmol/L) with a mean difference of 0.9 mmol/L (95% CI [−0.9, 2.7], d = 0.4, *p* = 0.35). The saturated fat content of the HGLP meals (mean: 5.8 ± 2.6 g) was significantly greater than the HGL meals (mean: 2.1 ± 0.5 g) with a mean difference of 3.8 g (95% CI [3.2, 4.4], *d* = 2.5, *p* < 0.001).

### 3.3. Glucose Outcome Measurements

There was no significant difference in the pre-prandial glucose, the insulin-to-carbohydrate ratio, or insulin dose timing between the control and any of the test meals (*p* > 0.05) (Supplementary Data, Table S7). The ingestion of the HGLP meal resulted in a significantly lower mean postprandial glucose (mean: 8.0 ± 2.2 mmol/L) when compared to the HGL meal (mean: 9.5 ± 2.5 mmol/L), with a mean difference of 1.6 mmol/L, 95% CI [0.7, 2.5], *d* = 0.7, *p* < 0.01). There were no other differences in the mean postprandial glucose between the meals.

There was no significant difference in the mean glucose excursion over the 180 min following ingestion of the control compared with the test meals (*p* > 0.05) (Supplementary Data, Table S7). The mean glucose excursion was significantly higher following the ingestion of the HGL meal (mean: 2.8 ± 2.5 mmol/L) compared with the HGLP meal (mean: 1.3 ± 1.8 mmol/L), with a mean difference of 1.6 mmol/L (95% CI [0.7, 2.5], *d* = 0.7, *p* < 0.01). There were no other significant differences in the glucose excursions between the test meals (Supplementary Data, Table S7).

The peak excursion was significantly higher following the ingestion of the HGL meal (mean: 6.4 ± 2.8 mmol/L) compared with the control meal (mean: 4.6 ± 2.9 mmol/L) with a mean difference of 1.9 mmol/L (95% CI [0.8, 3.0], *d* = 0.7, *p* < 0.01), the HGLP meal (mean: 4.2 ± 2.2 mmol/L) with a mean difference of 2.3 mmol/L (95% CI [1.2, 3.3], *d* = 0.9, *p* < 0.001) and the MGL meal (mean: 4.5 ± 2.6 mmol/L) with a mean difference of 2.1 mmol/L (95% CI [1.1, 3.1], *d* = 0.8, *p* < 0.01). There were no other significant differences in the peak excursion between the remaining meals (*p* > 0.05) (Supplementary Data, Table S7).

The time to peak (TTP) was significantly shorter following ingestion of the HGLP meal (mean: 58 ± 35 min) compared with the control (mean: 81 ± 45 min) with a mean difference of 24 min (95% CI [8, 41], *d* = 0.6, *p* = 0.03) and the MGL meal (mean: 96 ± 46 min) with a mean difference of 36 min (95% CI [22, 51], *d* = 1.0, *p* < 0.01). There were no significant differences in the TTP between the remaining meals (*p*> 0.05) (Supplementary Data, Table S7).

**Figure 2:**
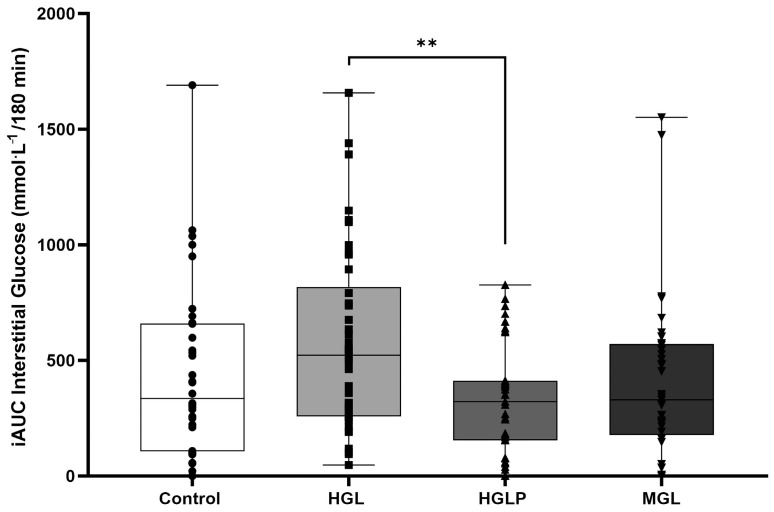
Incremental area under the interstitial glucose curve (iAUC) over the 3-h postprandial period following the ingestion of the control and test meals. HGL (high glycaemic load), HGLP (high glycaemic load with added protein), and MGL (moderate glycaemic load). Values are presented as median (IQR, range) ** indicates significant difference between meals, *p* < 0.01.

The glucose incremental area under the curve from 0 to 180 min (iAUC_0–180_) was significantly higher following ingestion of the HGL meals (mean: 579 ± 396 (mmol·min/L) compared with the HGLP meals (mean: 325 ± 242 (mmol·min/L), with a mean difference of 264 (mmol·min/L) (95% CI [126, 401], *d* = 0.8, *p* < 0.01). There was no significant difference in the iAUC between the remaining meals (*p* > 0.05) (Figure 2; Supplementary Data, Table S7).

The %CV was significantly higher after the ingestion of the HGL meals (mean: 26.4 ± 9.5%) compared with the control meals (mean: 19.7 ± 8.9%) with a mean difference of 6.9% (95% CI [3.3, 10.4], *d* = 0.8, *p* < 0.01) and the MGL meals (mean: 19.8 ± 6.4%) with a mean difference of 6.7% (95% CI [3.6, 9.8], *d* = 0.8, *p* < 0.01). There were no significant differences in the %CV between the remaining meals (*p* > 0.05) (Supplementary Data, Table S7).

### 3.4. Time in Range

The TIR (min) after ingestion of the HGL meal over 180 min (mean: 100 ± 56 min) was significantly shorter than after ingestion of the control meals (mean: 135 ± 50 min), with a mean difference of 35 min (95% CI [14, 56], *d* = 0.7, *p* = 0.01). This was also significantly shorter compared with the HGLP meals (Mean: 132 ± 53 min) with a mean difference of 33 min (95% CI [12, 54], *d* = 0.6, *p* = 0.02) and MGL meals (mean: 131 ± 49 min) with a mean difference of 31 min (95% CI [10, 52], *d* = 0.6, *p* = 0.03). There were no significant differences in the TIR (min) between the remaining meals (*p* > 0.05) (Supplementary Data, Table S7).

The TITR was significantly longer following ingestion of the HGLP meal (mean: 97 min ± 54 min) compared with the HGL meal (mean: 60 ± 46 min) with a mean difference of 38 min (95% CI [17, 58], *d* = 0.8, *p* < 0.01) and after the MGL meal (mean: 69 ± 50 min) with a mean difference of 29 min (95% CI [8, 50], *d* = 0.6, *p* = 0.04). There were no significant differences in the TITR (min) between the remaining meals (*p* > 0.05) (Supplementary Data, Table S7).

### 3.5. Glucose Excursions at 30-Min Time Intervals

**Figure 3:**
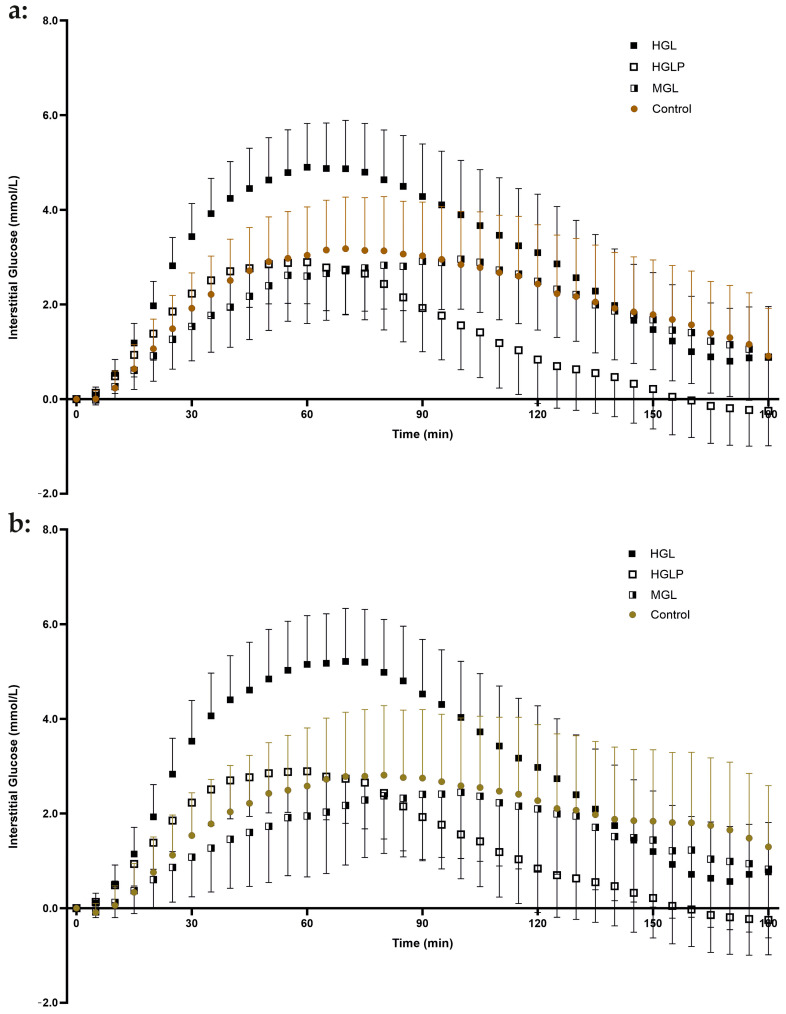
Mean Glucose Excursions over 180 min for 25 participants following consumption of the control and test breakfast meals. (**a**) All insulin regimens. (**b**) Participants using hybrid closed-loop (HCL) insulin delivery systems only. HGL (high glycaemic load), HGLP (high glycaemic load with added protein), and MGL (moderate glycaemic load). Error bars represent 95% CI.

Figure 3 presents the glucose excursions at each time point for breakfast meal type. Significant differences in the meal glucose excursions were apparent from 30 min after the meal. The mean glucose excursion was significantly greater following ingestion of the HGL meal at 30 min compared with the control (*p* < 0.001), the HGLP meal (*p* = 0.01), and the MGL meals (*p* < 0.001). By 60 min, the glucose excursion remained significantly higher after ingestion of the HGL meal compared with the control meals (*p* < 0.001), the HGLP meals (*p* < 0.001) and the MGL meals (*p* < 0.001) (Table 2a). At 90 and 120 min, the mean glucose excursion remained significantly higher after ingestion of the HGL meal compared with the HGLP meal (*p* = 0.01). There were no significant differences in the mean glucose excursion between the meals at 150 and 180 min (*p* > 0.05) (Table 2a). These results were comparable to those using HCL systems (Table 2b, Figure 3b).

### 3.6. Repeat Meal Reliability

Repeat meal reliability, assessed using intraclass correlation coefficients (ICCs) of iAUC data, was moderate for the control meal (ICC = 0.50) and for the HGL and HGLP meals (ICC = 0.60). In contrast, comparison of the incremental area under the curve (iAUC) for repeated MGL meals indicated poor reliability (ICC = 0.30).

### 3.7. Hypoglycaemic Events

There were thirty hypoglycaemic events within the 3-h postprandial period. Nine occurred following ingestion of the HGL meal, 8 in the HGLP meals, 7 for the MGL meals and 6 after ingestion of the control meals. There was no significant difference in the number of hypoglycaemia events between any of the meal conditions (*p* > 0.05).

## 4. Discussion

Consumption of the HGL test meal resulted in a significantly greater peak glucose excursion and reduced time spent within the target glucose range, compared with all other test meals. A key finding of the present study is that the addition of a protein food containing 10 g of protein and varying amounts of fat to an HGL meal resulted in a substantially improved postprandial glycaemic response with a similar glucose response to consumption of the MGL meal and with a higher level of repeat meal reliability than the MGL meals. These findings align with previous test-meal studies demonstrating that high-GI breakfasts elicit pronounced postprandial glucose rises in children and young people (CYP) with type 1 diabetes (T1D), particularly when compared with lower-GI meals [11,47]. The new evidence presented here indicates that the addition of a food containing 10 g of protein with varying amounts of fat to a high-GI breakfast meal is sufficient to make a meaningful difference in glycaemic control.

In direct comparison with the MGL breakfast, which had a low GI, the HGL meal produced a more rapid and greater initial glucose rise. Although rapid postprandial glucose increases following high-GI meals have been associated with postprandial hypoglycaemia [48], this was not observed in the present study, with no significant differences in hypoglycaemic events between meals. The iAUC following the protein-enriched high-GI meal (HGLP) was almost half that observed following the HGL meal and was comparable to that observed after ingestion of the low-GI MGL meal. Importantly, all assessed glycaemic outcome measures—including peak glucose, iAUC, and time in range—were significantly improved with the addition of the protein food. Time spent in the target range exceeded recommended thresholds, supporting recent ISPAD recommendations that encourage optimising postprandial glycaemia and time in range as key goals of T1D management.

From a clinical perspective, these findings reinforce current ISPAD guidance emphasising carbohydrate quality, including glycaemic index, as an important determinant of postprandial glycaemia. However, the current study findings indicate that incorporating 10 g of protein into high-GI breakfast meals could be an alternative to adopting lower-GI breakfast meal choices, representing a practical and sustainable strategy to improve postprandial glucose control and time in range in CYP with T1D, alongside carbohydrate counting and appropriate insulin dosing, without increasing hypoglycaemia risk.

Although overall postprandial glucose responses were similar following the ingestion of the HGLP and MGL meals, participants spent more time within the tight glucose target range after the HGLP meal. Repeat-meal reproducibility was also lower for the MGL breakfast than for the HGL and HGLP breakfasts, suggesting greater intra-individual variability in glycaemic responses to carbohydrate-containing meals. In contrast, protein ingestion has been shown to produce more predictable and physiologically constrained glucose responses in adults with T1D [20]. Accordingly, findings relating to the MGL meal should be interpreted cautiously, given its lower reproducibility and the consequent reduction in the reliability of between-meal comparisons.

Protein may influence postprandial glycaemia through several mechanisms, including delayed gastric emptying, stimulation of incretin hormones such as glucagon-like peptide-1 (GLP-1), and enhanced glucagon secretion [49,50]. In individuals with T1D, both dietary protein and fat have been shown to affect postprandial glycaemic excursions and insulin requirements independently of carbohydrate intake [13,14]. However, the lower glycaemic excursion observed following consumption of the HGLP breakfast meal in the present study is unlikely to be attributable to protein alone, as protein-containing foods also contain varying amounts of total and saturated fat. Consequently, the observed effect likely reflects the combined actions of protein and fat on postprandial glucose regulation.

Both dietary protein and fat have been shown to modulate gastric emptying and glucose metabolism. Dietary fat has been shown to delay gastric emptying in children and adolescents with T1D [51,52], thereby slowing the appearance of glucose in the circulation. Smart et al. (2013) [13] further demonstrated that a high-fat meal resulted in a lower early postprandial glucose excursion than a high-protein meal, suggesting that fat may exert a greater influence on the initial glycaemic response. In contrast, Faber et al. [21] reported that the addition of moderate amounts of both protein and fat reduced postprandial glucose excursions. Taken together, these findings suggest that the lower glycaemic response observed in the present study may be explained by the combined effects of protein and fat rather than by protein alone. This suggests that the inclusion of a modest portion of a food from the protein food group may represent a flexible and pragmatic dietary strategy to mitigate post-breakfast hyperglycaemia, particularly if lower-GI alternatives are deemed impractical or unacceptable for individuals. These findings extend existing evidence and provide empirical support for ISPAD guidance recommending protein enrichment of breakfast meals known to elicit postprandial hyperglycaemia.

High-protein and high-fat meals have previously been associated with delayed postprandial hyperglycaemia, particularly when intake exceeds 40 g, although definitions of “high protein/fat” remain inconsistent due to substantial inter-individual variability [1]. In the present study, the addition of 10 g of protein (resulting in a mean total protein intake of 18.5 ± 1.6 g) did not result in late postprandial hyperglycaemia within the 3-h observation period. This is consistent with findings by Paterson et al. (2017) [14], who reported glucose increases of approximately 0.27–0.64 mmol/L per 10 g protein between 150 and 300 min, and observed minimal absolute excursions at similar protein intakes, although this was with negligible amounts of fat. Smart et al. (2013) [13] demonstrated delayed hyperglycaemia beyond 180 min following high-fat, high-protein meals which contained substantially higher amounts of protein (≥40 g) and fat (>35 g) than in the present study. In contrast, none of the HGLP meals in the present study contained more than 20 g fat, which may partially explain the absence of delayed hyperglycaemia over the three-hour postprandial period. However, as delayed hyperglycaemia can occur many hours after the consumption of meals containing fat and protein, it is not possible to make conclusions about whether this may have occurred in this study had a longer postprandial period been observed.

Collectively, these findings support ISPAD guidance advocating protein inclusion in breakfast meals known to cause postprandial hyperglycaemia and provide preliminary evidence that modest protein and fat enrichment may be sufficient to improve postprandial glycaemia without increasing short-term risk of delayed hyperglycaemia. Whether similar effects persist beyond three hours remains uncertain. However, the three-hour postprandial period reflects typical mid-morning snack timing in the UK, at which point insulin adjustment affords an opportunity to address emerging hyperglycaemia. Delayed hyperglycaemia beyond three hours may therefore be of less clinical significance following breakfast than following the evening meal, which is often the final meal before sleep—a distinction previously highlighted by Smart et al. (2013) [13].

Despite the glycaemic benefits observed, an important nutritional consideration is that protein addition significantly increased the saturated fat and salt content of the HGLP meals. Some commonly chosen protein foods such as cheese, bacon, and sausages are high in saturated fat and were frequently selected in our previous observational work [25]. Given the elevated cardiovascular risk associated with T1D, dietary strategies should prioritise protein sources low in saturated fat. In the present study, the choice of protein sources for the HGLP meals reflected typical dietary behaviour. However, since amino acid composition and absorption rate determine the postprandial glycaemic response [53] this might have impacted the glycaemic response. Indeed, in healthy participants significant differences in glycaemic response and insulin secretion have been observed following the ingestion of different protein sources [54,55]. However, in a recent study including adults with T1D no significant differences in glycaemic outcomes between common protein sources were observed [20], suggesting that clinical recommendations need not differentiate by protein type for this population. Nevertheless, the potential influence of protein source warrants further investigation in larger, controlled studies and should explore the acceptability and glycaemic effects of lean protein options in this context.

In addition, the present study has shown that significant postprandial hyperglycaemia following the high-GI breakfast can still occur despite HCL systems use, reinforcing the limitations of current automated insulin delivery in responding to rapid glucose absorption. Managing postprandial glycaemia remains challenging even in individuals using HCL insulin delivery systems [56], and time above range in HCL users is largely attributable to postprandial hyperglycaemia, particularly after breakfast [57,58,59]. While some studies suggest HCL systems can attenuate glycaemic variability following high-protein meals [59], others show that high glycaemic load remains a strong predictor of reduced time in range [60]. These findings highlight the continued importance of dietary strategies to complement technological advances in insulin delivery.

Finally, the pragmatic design of this study necessitated unsupervised meal consumption and reliance on self-reported dietary intake. Consequently, food intake could not be directly controlled, and deviations from the study protocol, including inaccuracies in the reporting of portion sizes, cannot be excluded. The use of three different portion sizes may have further increased the potential for reporting error and contributed to greater variability in postprandial glycaemic outcomes. Although the use of weighed meals and photographic recording of food intake may have improved dietary assessment, these measures could have increased participant burden and potentially adversely affected recruitment and retention. Future studies should therefore consider standardising portion sizes to reduce variability and improve the precision of estimates of macronutrient effects on postprandial glycaemia. While the pragmatic nature of the study enhances validity and real-world applicability, it inevitably limits dietary standardisation and constrains causal inference. Furthermore, the sample size was modest, and for effect sizes below 0.64 the study may be underpowered, although several outcomes exceeded this threshold. Furthermore, our participants demonstrated relatively good glycaemic control at baseline, with mean HbA1c below ISPAD targets, and inclusion was limited to users of Dexcom CGM, potentially introducing selection bias.

## 5. Conclusions

This pragmatic study demonstrates that the addition of a protein food with fat providing 10 g of protein to a high glycaemic index breakfast meal can substantially improve postprandial glycaemic outcomes in children and young people with type 1 diabetes, without inducing short-term delayed hyperglycaemia. These findings have important practical relevance for nutritional guidance and support modest protein enrichment as a flexible and achievable strategy to mitigate post-breakfast glycaemic excursions in real-world settings, including among individuals using hybrid closed-loop systems.

The nutritional quality of protein sources warrants careful consideration. Given the increased saturated fat and salt content associated with some commonly chosen protein foods, dietary advice should preferentially promote lower-fat, lower-salt protein options, particularly in this population with elevated long-term cardiovascular risk. Further research is required to confirm these findings in larger and more diverse cohorts, to evaluate longer-term effects on overall glycaemic control, and to assess the acceptability and feasibility of incorporating less processed protein sources into breakfast meals for children and young people with type 1 diabetes.

## Figures and Tables

**Table 1:** Clinical characteristics of participants.

Characteristics	
Sex	
Male	15
Female	10
Insulin pump therapy	23
Multiple daily injection therapy	2
Age (years)	12.1 ± 3.6
Duration of diabetes (years)	4.3 ± 3.2
BMI z score	0.42 (1.6)
HbA1c, mmol/mol	48.4 ± 5.4
Type of Insulin	
Aspart (NovoRapid)	21
Lispro (Humalog)	1
Aspart (Fiasp)	3
Type of insulin pump therapy	
Open	6
Hybrid Closed Loop	17
Insulin-to-carbohydrate ratio (units/g)	1:11 ± 5.3

Parametric data: mean ± SD, non-parametric data: median (IQR).

**Table 2:** (a) Mean Glucose Excursions by meal type for all regimens at 30 min intervals to 180 min. (b). Mean Glucose Excursions by meal type for HCL system at 30 min intervals to 180 min.

	Mean Postprandial Glucose Excursion (mmol/L)			
Minutes After the Meal	Control	HGL	HGLP	MGL
(a)				
30	1.9 (1.2–2.6)	3.4 (2.8–4.1) ^a,b,c^	2.2 (1.6–2.9)	1.5 (0.8–2.2)
60	3.0 (2.1–4.0)	4.9 (4.0–5.8) ^a,b,c^	2.9 (2.0–3.7)	2.6 (1.6–3.6)
90	3.0 (1.9–4.1)	4.3 (3.2–5.4) ^b^	1.9 (1.0–2.8)	2.9 (1.9–3.9)
120	2.4 (1.2–3.6)	3.1 (1.9–4.3) ^b^	0.8 (−0.1–1.7)	2.5 (1.5–3.5)
150	1.8 (0.7–2.9)	1.5 (0.3–2.6)	0.2 (−0.6–1.0)	1.7 (0.7–2.7)
180	0.9 (−0.1–1.9)	0.9 (−0.1–1.9)	−0.3 (−1.0–0.5)	0.9 (−0.1–1.9)
(b)				
30	1.5 (0.7–2.4)	3.5 (2.7–4.4) ^a,b,c^	2.0 (1.1–2.9)	1.1 (0.3–1.9)
60	2.6 (1.4–3.8)	5.2 (4.2–6.1) ^a,b,c^	2.6 (1.6–3.6)	2.0 (0.7–3.2)
90	2.8 (1.4–4.1)	4.5 (3.4–5.6) ^b,c^	1.7 (0.6–2.8)	2.4 (1.1–3.7)
120	2.3 (0.7–3.8)	3.0 (1.7–4.2) ^b^	0.8 (−0.3–2.0)	2.1 (0.9–3.3)
150	1.8 (0.4–3.3)	1.2 (0.0–2.4)	0.3 (−0.7–1.3)	1.4 (0.1–2.8)
180	1.3 (0.1–2.5)	0.8 (−0.2–1.8)	−0.2 (−1.2–0.7)	0.8 (−0.5–2.2)

Mean glucose excursion (95% CI) ^a,b,c^ statistically different excursion of HGL meal compared with control, HGLP and MGL meals respectively (*p* ≤ 0.01).

## Data Availability

The data to support these findings are available from the corresponding author on request.

## Supplementary Materials

The following supporting information can be downloaded at: https://www.mdpi.com/article/10.3390/nu18152536/s1, Table S1: Contents of the HGL meal; Table S2: Contents of the HGLP meal; Table S3: Contents of the MGL breakfast meal; Table S4: The type of cereal and the portion size participants chose for the HGL and HGLP breakfast meals; Table S5: The type of protein foods chosen by participants for the HGLP breakfast meal; Table S6: The nutrient composition of each of the control and test meals for the 25 participants, with comparisons made between the control and test meals after averaging the repeated meals; Table S7: The pre-prandial glucose, insulin dose timing, and postprandial glucose measurements from CGM readings taken over 180 min following ingestion of each type of meal, with comparisons between control and each test meal.

## Abbreviations

The following abbreviations are used in this manuscript:

GI	Glycaemic Index
CYP	Children and Young People
T1D	Type 1 Diabetes
GL	Glycaemic Load
HGL	High Glycaemic Load
HGLP	High Glycaemic Load Protein
MGL	Medium Glycaemic Load
HCL	Hybrid Closed-Loop
TIR	Time in Range
ISPAD	International Society of Paediatric and Adolescent Diabetes
RCT	Randomised Control Trial
MDI	Multiple Daily Injections
CSII	Continuous Subcutaneous Insulin Infusion
CGM	Continuous Glucose Monitoring
BMI	Body Mass Index
HbA1C	Glycated Haemoglobin
iAUC	Incremental Area Under the Curve
CV	Coefficient of Variation
TBR	Time below Range
TITR	Time in Tight Range
TAR	Time above Range
SD	Standard Deviation
IQR	Interquartile range
REML	Restricted Maximum Likelihood
ICC	Intraclass Correlation Coefficient
CI	Confidence Interval
