# Supplementary material for: Protein-Containing Breakfasts Are Associated with Reduced Postprandial Glycaemic Excursions in Children and Young People with Type 1 Diabetes: A Pragmatic Study
Source: Nutrients. 2026 Aug 4;18(15):2536. doi: 10.3390/nu18152536 (PMC13467609; doi:10.3390/nu18152536)
Supplement: Supplementary file 1 [file nutrients-18-02536-s001.zip › nutrients-4429753-supplementary.pdf]

Table S1. Contents of the HGL meal

Meal contents	Carbohydrate (g) of portion size		
	35 g	50 g	70 g
Rice based cereal or corn flakes cereal	35g	50g	70g
Milk semi skimmed	150ml	200ml	250ml
Drink	250-500ml water or sugar free diluting juice		

Table S2. Contents of the HGLP meal

Meal content	Carbohydrate (g) of portion size		
	35 g	50 g	70 g
Rice based cereal or corn flakes cereal	35g	50g	70g
Milk semi skimmed	150ml	200ml	250ml
Drink	250-500ml water or sugar free diluting juice		
Plus **one** of the following	One average size egg (e.g. boiled or scrambed with no milk) and 15g hard cheese<br>One average size egg (e.g. boiled or scrambed with no milk) and two thin slices of ham (22g<br>One average size egg (e.g. boiled or scrambed with no milk) and 15 g rasher of bacon<br>40 g hard cheese e.g. cheddar<br>55g of wafer thin ham<br>40 g bacon<br>45 g tinned fish<br>45 g cooked chicken<br>50 g mackerel fillet		

Table S3. Contents of the MGL breakfast meal

**MGL meal contents**
One medium slice of bread, with added fibre e.g. *Kingsmill 50:50 medium sliced loaf, Hovis Best of Both medium sliced loaf, Warburtons Half and Half medium sliced loaf*<br><br>With<br>10g chocolate and nut spread e.g. *Nutella* or a supermarket version of Nutella<br><br>And<br>150g of full fat natural yoghurt e.g. *Yeo Valley Natural Yoghurt* or supermarket own version<br><br>With 80g of Mixed berries<br><br>The mixed berries:<br>Either 2 from the following:<br>40g Strawberries<br>40g Raspberries<br>40g Blueberries<br>40g Blackberries<br><br>Or 20g of each<br>20g Strawberries<br>20g Raspberries<br>20g Blueberries<br>20g Blackberries<br><br>Or 80g of frozen berries – defrosted<br>Drink: 250-500ml water or sugar free diluting juice

Table S4. The type of cereal and the portion size participants chose for the HGL and HGLP breakfast meals

	HGL meals	HGLP meals
Rice cereal	24 (3)	20 (2)
Corn cereal	18 (3)	14 (1)
Type of cereal unknown	n/a	2 (3)
35 g	24 (4)	23 (2)
50 g	15 (1)	12 (2)
70 g	3 (1)	1 (2)

n = number meals included (number excluded). Portion size = grams of carbohydrate

Table S5. The type of protein foods chosen by participants for the HGLP breakfast meal.

**Protein food**	**n**
Egg and cheese	8 (2)
Bacon	9
Cheese	5 (4)
Egg and ham	4
Egg and bacon	4
Chicken	3
Ham	1
Frankfurter	1
Egg only	1

n = number meals included (number excluded)

Table S6. The nutrient composition of each of the control and test meals for the 25 participants with comparisons made between the control and test meals after averaging the repeated meals.

	**Control**	**HGL**	**HGLP**	**MGL**
Energy (kcal)	372 ± 147	242 ± 55[a]	351 ± 61[b]	307 ± 0[abc]
Fat (g)	13.5 ± 10.5	3.5 ± 1.0[a]	12.1 ± 4.0[b]	8.9 ± 0.0[ab]
SFA (g)	5.5 ± 4.5	2.1 ± 0.5[a]	5.8 ± 2.6[b]	4.0 ± 0.0[bc]
CHO (g)	48.0 ± 20.7	43.6 ± 10.2	42.0 ± 8.5[a]	39.6 ± 0.0[a]
Sugars (g)	20.6 ± 10.3	16.0 ± 5.3[a]	15.0 ± 4.3[a]	23.2 ± 0.0[bc]
Fibre (g)	4.7 ± 5.1	0.7 ± 0.4[a]	0.7 ± 0.3[a]	5.9 ± 0.0[bc]
Protein (g)	13.0 ± 10.5	8.8 ± 2.1[a]	18.5 ± 1.6[ab]	13.6 ± 0.0[bc]
Salt (g)	0.7 ± 0.3	0.7 ± 0.2	1.4 ± 0.4[ab]	0.7 ± 0.0[c]
GI%	60 ± 11	72 ± 1[a]	72 ± 1[a]	41 ± 0[abc]
GL	29 ± 15	32 ± 8	30 ± 6	16 ± 0[abc]

Mean ± SD. Linear Mixed Model fit by REML with fixed intercept and random intercepts (participants). Number of meals: control (n=32), HGL (n=42), HGLP (n=36), MGL meals (n=37). Normality of residuals tested using skewness and Kurtosis. [a, b, c]: $p<0.05$ compared with Control, HGL and HGLP, respectively.

Table S7. The pre-prandial glucose, insulin dose timing and postprandial glucose measurements from CGM readings taken over 180min following ingestion of each type of meal with comparisons between control and each test meal.

	**Control**	**HGL**	**HGLP**	**MGL**
Pre-prandial glucose (mmol/l)	6.8 ± 1.4	6.9 ± 1.3	6.9 ± 1.3	7.1 ± 1.3
Insulin: carbohydrate ratio	11.0 ± 4.9	10.6 ± 6.0	10.3 ± 5.3	10.8 ± 5.2
Insulin dose lag time (min)	13.4 ± 7.9	14.0 ± 8.6	12.2 ± 8.1	12.4 ± 8.4
Mean PPG (mmol/l)	8.4 ± 2.6	9.5 ± 2.5	8.0 ± 2.2[b]	8.7 ± 2.2
Mean excursion (mmol/l)	2.1 ± 2.4	2.8 ± 2.5	1.3 ± 1.8[b]	1.9 ± 2.3
Peak glucose excursion (mmol/l)	4.6 ± 2.9	6.4 ± 2.8[a]	4.2 ± 2.2[b]	4.5 ± 2.6[b]
Time to peak (min)	81 ± 45	72 ± 31	58 ± 35[a]	96 ± 46[c]
AUC (mmol/l/180min)	440 ± 379	579 ± 396	325 ± 242[b]	408 ± 348
%CV	19.7 ± 8.9	26.4 ± 9.5[a]	23.0 ± 8.0	19.8 ± 6.4[b]
TIR (min)	135 ± 50	100 ± 56[a]	132 ± 53[b]	131 ± 49[b]
TAR (min)	41 ± 50	74 ± 57[a]	45 ± 54[b]	47 ± 50
TITR (min)	88 ± 58	60 ± 46	97 ± 54[b]	69 ± 50[c]

Mean ± SD. Linear Mixed Model fit by REML with fixed intercept and random intercepts (participants). Number of meals: control (n=32), HGL (n=42), HGLP (n=36), MGL meals (n=37). Normality of residuals tested using skewness and Kurtosis. [a, b, c]: $p<0.05$ compared with Control, HGL and HGLP, respectively.
